# A genetic algorithm-based framework for online sparse feature selection in data streams

**DOI:** 10.3389/fdata.2026.1782461

**Published:** 2026-02-09

**Authors:** Guanyu Liu, Jinhang Liu, Guifan He, Yifan Liu, Huabo Bai, Min Zhou

**Affiliations:** 1 College of Computer and Information Science, Southwest University, Chongqing, China; 2 PetroChina Qinghai Oilfield Company, Qinghai, China; 3 Office of Informatization Construction, Southwest University, Chongqing, China

**Keywords:** feature selection, genetic algorithm, latent factor analysis, missing data, online learning

## Abstract

High-dimensional streaming data implementations commonly utilize online streaming feature selection (OSFS) techniques. In practice, however, incomplete data due to equipment failures and technical constraints often poses a significant challenge. Online Sparse Streaming Feature Selection (OS^2^FS) tackles this issue by performing missing data imputation via latent factor analysis. Nevertheless, existing OS^2^FS approaches exhibit considerable limitations in feature evaluation, resulting in degraded performance. To address these shortcomings, this paper introduces a novel genetic algorithm-based online sparse streaming feature selection (GA-OS^2^FS) in data streams, which integrates two key innovations: (1) imputation of missing values using a latent factor analysis model, and (2) application of genetic algorithm to assess feature importance. Comprehensive experiments conducted on six real-world datasets show that GA-OS^2^FS surpasses state-of-the-art OSFS and OS^2^FS methods, consistently attaining higher accuracy through the selection of optimal feature subsets.

## 1 Introduction

The rapid advancement of information technology has led to the widespread generation of high-dimensional data characterized by multiple levels, granularities, and modalities. This complexity poses significant challenges to foundational technologies in fields such as artificial intelligence, data management, communication, and storage (Yao et al., 2022; Ramírez-Gallego et al., 2018; Li Z. et al., 2025). To address the issues associated with high-dimensional data, feature selection has proven to be a highly effective technique (Chandrashekar and Sahin, 2014; Casmiry et al., 2025; Wang et al., 2025; Chen et al., 2025). In recent years, a diverse array of feature selection methodologies has emerged (Kundu and Mitra, 2017; Yang et al., 2018; Albattah and Khan, 2025), which can be broadly categorized into filter-based, wrapper-based (Xue et al., 2018), and embedded approaches (Xue et al., 2016). Furthermore, in the context of big data applications, the feature space frequently expands dynamically, potentially to an infinite scale (Ni et al., 2017; Ditzler et al., 2018). This reality has driven the development of Online Streaming Feature Selection (OSFS). For example, Wu et al. (2013) pioneered an OSFS framework utilizing online relevance and redundancy analysis. Their model classifies incoming features into strongly relevant, weakly relevant, and irrelevant groups, ultimately selecting features that are relevant (strongly or weakly) and non-redundant. Subsequently, Yu et al. (2016) introduced the SAOLA model, which extends this concept by evaluating the pairwise relationships between streaming features through a specific mechanism.

However, most existing Online Streaming Feature Selection (OSFS) models are formulated under the assumption of complete feature streams, where all incoming data points are fully observed without any missing values. In real-world scenarios, this assumption often fails to hold, as streaming features frequently contain substantial missing data due to a range of unforeseen factors. For instance, in single-cell sequencing, technological constraints make it challenging to profile every cell comprehensively, preventing reliable weight assignment for all measured entities (Badsha et al., 2020). Similarly, in clinical settings, complete patient data collection is often hindered by equipment failures or procedural inconsistencies (Idri et al., 2018). This prevalent issue gives rise to the challenge of Online Sparse Streaming Feature Selection (OS^2^FS), which addresses the critical question of how to reliably select features from a stream that is inherently sparse and contains significant missing entries.

In real-world recommendation systems, features—including user behavior logs and product attributes–are often received as continuous streams. Since users typically interact with only a fraction of available items, missing data is commonplace. These features are also highly interdependent, complicating the decision of which should be retained or removed to fully and precisely model user interests. Consequently, identifying the most representative feature subset is essential for delivering prompt and relevant recommendations. Traditional approaches to feature evaluation are largely designed for fully observed feature streams and tend to overlook the inaccuracies arising from the imputation of missing data. Such neglect is particularly consequential, as feature selection constitutes an NP-hard binary discrete optimization problem. Evolutionary computation (EC) techniques are notably effective in overcoming these challenges, providing robust solutions for problems of high combinatorial complexity. The core advantages of genetic algorithms lie in their global exploration capability, low dependency on problem assumptions, coding flexibility, and ease of parallelization, making them particularly suitable for complex optimization problems. Furthermore, its strong global search ability helps prevent convergence to local optima, increasing the likelihood of discovering feature subsets that optimize the trade-off between model accuracy and feature sparsity. These advantages have led to the widespread adoption of GA-based strategies in feature subset selection tasks. Therefore, this paper proposes a novel genetic algorithm-based online sparse streaming feature selection (GA-OS^2^FS) in data streams. In smart factories, GA-OS^2^FS processes incomplete, high-dimensional streaming data from sensors (e.g., vibration, temperature) by imputing missing values via latent factor analysis and dynamically selecting the most discriminative features (e.g., failure-indicative patterns) using a genetic algorithm. This enables real-time, accurate anomaly detection and predictive maintenance, minimizing unplanned downtime. For IoT-based energy management systems, GA-OS^2^FS handles sparse and irregular streaming data from distributed sensors (e.g., occupancy, temperature). It recovers missing values and employs genetic algorithm-based evaluation to identify and retain features most relevant to energy consumption. This results in an optimized feature subset for real-time control of HVAC and lighting systems, enhancing energy efficiency in smart buildings.

## 2 Related work

Online Streaming Feature Selection (OSFS) models, which process feature streams in real time, have garnered significant research interest. For instance, Perkins and Theiler (2003) introduced Grafting, a regularized online feature selection framework. However, it requires careful tuning of regularization parameters prior to feature selection, making it less adaptable to scenarios with an unknown or expanding feature space. Zhou et al. (2006) proposed the Alpha-investing strategy, capable of handling infinite feature streams, though it does not account for redundancy among the selected features. Wu et al. (2013) categorized incoming features into strongly relevant, weakly relevant, and irrelevant groups, developing two OSFS variants: OSFS and Fast-OSFS. The latter specifically addresses redundancy between newly arrived features and the already selected subset. Building on mutual information, Yu et al. (2016) presented the SAOLA model, which evaluates feature relevance based on pairwise interactions. To capture more complex dependencies, Zhou et al. (2021b) developed the OGSFS-FI model by examining interactions between feature groups. This was later extended to the SFS-FI model Zhou et al. (2021a), which can identify features involved in multi-way interactions, including two-way, three-way, and higher-order relationships. Furthermore, to better model dynamic decision-making, Zhou et al. (2022) applied the three-way decision (3WD) principle to propose the OSSFS-DD model, which computes partition thresholds according to 3WD theory to mitigate decision risk.

In parallel, rough set theory has proven to be a valuable framework for Online Streaming Feature Selection (OSFS). For example, Zhou et al. (2019b) introduced the OFS-A3M model, which employs a neighborhood rough set relation with adaptive neighbors to identify features that exhibit high relevance, strong dependency, and low redundancy. This work was later extended to the OFS-Density model (Zhou et al., 2019a), where a novel adaptive density-based neighborhood relation is used to analyze domain characteristics and configure model parameters. In a different approach, Luo et al. (2023) leveraged the concept of rough hypercuboids to develop the RHDOFS model. Similarly, Shu et al. (2024) proposed the ANOHFS model, which relies on an adaptive neighborhood mechanism to effectively identify closely related feature hierarchies within high-dimensional data. Zhuo et al. (2024) proposed an online feature selection method for dynamic feature spaces, with innovations in Gaussian Copula-based correlation modeling, real-time tree-ensemble selection, and geometric inference for unlabeled data. Qiu et al. (2025) proposed an online confidence learning algorithm for noisy labeled features. It tackles instance distribution shifts and label noise in data streams by employing online confidence inference and geometric structure learning. Although current OSFS models play a crucial role in dynamically selecting streaming features, to our knowledge, they still lack the ability to effectively handle sparse streaming features. Missing data tends to raise the computational cost of OSFS models and may also lead to the selection of less relevant or redundant features. Sparse streaming features often exhibit weak associations with other features or the target variable, complicating the reliable evaluation of their importance. Moreover, they can cause uneven data distributions, where certain sample values appear very rarely–a situation that may undermine the overall performance of OSFS. While these methods demonstrate considerable effectiveness in tackling conventional OSFS problems, they share a common limitation: all are designed under the assumption of complete feature streams and do not account for missing data, thus leaving the challenges of OS^2^FS scenarios unaddressed.

Latent factor analysis (LFA) has established itself as an effective approach for estimating missing data (Wu et al., 2022). The method operates by mapping the observed entries of a high-dimensional, incomplete matrix onto latent representations associated with its rows and columns (Zhang Z. et al., 2017; Zhang J. D. et al., 2017). A learning objective is formulated to quantify the discrepancy between the original observed values and their reconstructions (Luo et al., 2018; Gong et al., 2018). Subsequently, the model constructs a complete, low-rank approximation of the target incomplete matrix by minimizing this generalized error, as defined by the learning objective (Luo et al., 2021b; Wu et al., 2021).

## 3 Preliminaries

### 3.1 Online streaming feature selection

The Online Streaming Feature Selection (OSFS) model provides an effective approach for identifying the optimal subset of streaming features, which is accomplished through online relevance analysis and online redundancy analysis. Consider a streaming feature set *F* = {*F*_1_, *F*_2_, ..., *F*_*T*_} and a label set $C={\left[c_{1},c_{2},...,c_{M}\right]}^{\text{T}}$, where each feature $F_{t}={\left[f_{1,t},f_{2,t},...,f_{M,t}\right]}^{T}$ contains *M* samples, with *t* ∈ 1, 2, ..., *T*.

Suppose that two features *F*_*p*_ and *F*_*q*_, where *p* ≠ *q*, *p, q* ∈ 1, 2, ..., *T*, if *P*(*F*_*p*_|*F*_*q*_, *X*) = *P*(*F*_*p*_, *X*) or *P*(*F*_*p*_|*F*_*q*_, *X*) = *P*(*F*_*q*_, *X*), such that *F*_*p*_ and *F*_*q*_ are conditionally independent to the subset *X* ⊆ *F*.

For a streaming feature *F*_*t*_ at the time stamp *t*,

a) if ∀ς ⊆ *F* − *F*_*t*_ s.t. *P*(*C*|ς, *F*_*t*_) ≠ *P*(*C*|ς), then decide *F*_*t*_ is strong relevance;
b) if ∃ς ⊆ *F* − *F*_*t*_ s.t. *P*(*C*|ς, *F*_*t*_) ≠ *P*(*C*|ς), then decide *F*_*t*_ is weak relevance;
c) if ∀ς ⊆ *F* − *F*_*t*_ s.t. *P*(*C*|ς, *F*_*t*_) = *P*(*C*|ς), then decide *F*_*t*_ is irrelevance.

Given a relevant feature *F*_*t*_(*M*(*F*_*t*_) ∉ *M*(*C*)_*t*_), and the redundant set *X*_*F*_ is denoted as follows:


$$\begin{array}{l} \begin{array}{l} \forall X_{F}\in M{\left(C\right)}_{t}\cup F_{t}\;,\exists \zeta \subseteq M{\left(C\right)}_{t}\cup F_{t}\;-\left\{X_{F}\right\}\\ \text{s.t.}\;P\left(C|X_{F},\zeta \right)=P\left(C|\zeta \right) \end{array} \end{array}$$ (1)

where *M*(·) denotes Markov blanket.

### 3.2 Latent factor analysis

The Latent Factor Analysis (LFA) model plays a significant role in pre-estimating sparse matrices. This section begins by presenting the formal definition of the LFA model (Hancer et al., 2022; Wang et al., 2023).

Let R^*M* × *H*^ be a sparse matrix, and an LFA model trains two latent factor matrices *U*^*M* × *L*^ and *V*^*H* × *L*^ via the know entries, which precisely represent the rank-*L* approximation $\hat{R}$ of R, where $\hat{R}$ is formulated as $\hat{R}$ = *UV*^T^, *L* is the dimension of *U* and *V*, and *L* ≪ min|*M*|, |*H*| (Li et al., 2024; Hancer et al., 2025).

The error of the LFA model is then formulated as:


$$\begin{array}{l} E\left(U,V\right)=\underset{r_{m,h}\in \Lambda }{\sum }e\left(\Delta m.h\right),\Delta m,h=rm,h-\hat{r}m,h\left(u_{m},v_{h}\right), \end{array}$$ (2)

where Λ denotes the known data of R, *e*(·) calculates the error between *r*_*m, h*_ and ${\hat{r}}_{m,h}$, *r*_*m, h*_ is *m*-th row and *h*-th column of R, the ${\hat{r}}_{m,h}$ is the predicted value for *r*_*m, h*_, ${\hat{r}}_{m,h}\left(\cdot \right)$ stands for predictive function.

Incorporating regularization is essential for the LFA model to prevent over-fitting (Wu et al., 2023; Li et al., 2023). Thus, by integrating regularization into Equation 2, the following objective function is derived:


$$\begin{array}{l} \begin{array}{l} \varepsilon \left(U,V\right)=\underset{r_{m,h}\in \Lambda }{\sum }e\left(r_{m,h}-{\hat{r}}_{m,h}\left(u_{m},v_{h}\right)\right)\;+\frac{\lambda }{2}\left(|U{|}_{F}^{2}+|V{|}_{F}^{2}\right) \end{array}, \end{array}$$ (3)

where |·|_*F*_ computes the Frobenius norm, λ represents the regularization coefficient.

## 4 Proposed algorithm

### 4.1 Problem of GA-OS^2^FS

Consider a collection of sparse streaming features denoted by $F^{\prime }=\left\{F_{1}^{\prime },F_{2}^{\prime },...,F_{T}^{\prime }\right\}$, which is postulated to possess a missing data rate of ρ. Here, ρ = 1 − |Λ|/*M*, with |·| representing the cardinality of a set. From time point *t* to *t* + *H*-1, sparse streaming features *F*′*t, F*′*t* + 1, ..., *F*′*t* + *H* − 1 are generated sequentially and collected into an R^*M* × *H*^ buffer. This buffer, of size *H*, forms the sparse streaming feature matrix ${\text{B}}_{t}=\left\{F_{t}^{\prime },F_{t+1}^{\prime },...,F_{t+H-1}^{\prime }\right\}$. Subsequently, a completed streaming feature matrix, expressed as ${\hat{B}}_{t}=\left\{{\hat{F}}_{t}^{\prime },{\hat{F}}_{t+1}^{\prime },...,{\hat{F}}_{t+H-1}^{\prime }\right\}$, is estimated based on the observed known data.

The principal objective of the GA-OS^2^FS method is to identify the optimal feature subset. Consequently, the GA-OS^2^FS framework is designed to address the following optimization problem:


$$\begin{array}{l} S_{t}=\underset{\sigma \subseteq S_{t}}{\arg\max}P\left(C|\sigma \right). \end{array}$$ (4)

### 4.2 The framework of GA-OS^2^FS

The framework of GA-OS^2^FO consists of two steps: first, estimating missing values, and then assessing feature importance.

#### 4.2.1 Estimate sparse streaming features in advance

In practical applications, data quality is often difficult to guarantee due to missing feature values, making it exceptionally challenging to screen high-quality features from feature streams. Taking a medical monitoring system as an example, if a sensor fails and causes data loss, traditional OSFS models may transmit erroneous signals to control devices, which could ultimately endanger patients' lives. Therefore, preprocessing the data before feature selection to impute missing entries is of crucial importance. The LFA model holds significant value for missing-data imputation, as it completes missing values by mapping the sparse matrix onto two latent factor matrices (Li J. et al., 2025; Chen J. et al., 2024; Yuan et al., 2025). Traditional methods—such as mean imputation and matrix factorization—typically fill missing values based on observed data and rely on assumptions such as linearity or local similarity, which limits their ability to capture complex non-linear relationships in high-dimensional or sparse streaming data (Chen et al., 2023; Xu et al., 2025b). In contrast, the LFA model can capture the underlying structure of the data through latent space modeling, thereby handling complex dependencies and non-linear patterns more effectively (Liao et al., 2025; Lin M. et al., 2025).

The complete latent features extracted from incomplete data can be used for missing-value imputation, classification, clustering, and other tasks (Wu et al., 2025b; Lyu et al., 2026). The extraction methods are mainly divided into linear and non-linear feature extraction. Linear feature extraction mostly employs LFA-based models that rely on matrix factorization. When dealing with sparse data, such models aim to construct a low-rank approximation of the high-dimensional incomplete matrix (Wu et al., 2024; Wu H. et al., 2025). They map the known entries of the target high-dimensional incomplete matrix to its row and column nodes, formulate a learning objective that measures the discrepancy between the actual data and the estimated data, and thereby generate a complete low-rank approximation matrix of the target high-dimensional incomplete matrix. An optimizer is then used to minimize linear error, achieving efficient representation (Wu et al., 2025a; Qin et al., 2024; Xu et al., 2025a; Chen M. et al., 2024).

The initialization procedure subjects both matrices *U* and *V* to small random values. These values, generated by scaling a random permutation to a vicinity close to zero, serve as the non-zero starting point for the iterative algorithm, for which initial conditions are crucial. The following illustration details the update method, taking matrix *U* as a representative case.


$$\begin{array}{l} u_{m,k}=0.004-0.004\times \frac{randperm\left(1,000,1\right)}{1,000}. \end{array}$$ (5)

The LFA model constructs a low-rank approximation for *R* (Tang et al., 2024; Luo et al., 2021a). Typically, matrices *U* and *V* are derived from *R* by minimizing a loss function defined by the Euclidean distance between *R* and $\hat{R}$ (Xu et al., 2023). Building upon Equations 2, 3, the complete streaming features are predicted using the known values according to:


$$\begin{array}{l} \varepsilon =\underset{f_{m,j}^{\prime }\in \Lambda }{\sum }\left(\frac{1}{2}{\left(f_{m,j}^{\prime }-\overset{L}{\underset{k=1}{\sum }}u_{m,k}v_{j,k}\right)}^{2}+\frac{\lambda }{2}\left(\overset{L}{\underset{k=1}{\sum }}u_{m,k}^{2}+\overset{L}{\underset{k=1}{\sum }}v_{j,k}^{2}\right)\right). \end{array}$$ (6)

Subsequently, the loss function for the *m*-th element *f*_*m, j*_ is calculated as:


$$\begin{array}{l} \varepsilon_{m,j}=\frac{1}{2}{\left(f_{m,j}^{\prime }-\overset{L}{\underset{k=1}{\sum }}u_{m,k}v_{j,k}\right)}^{2}+\frac{\lambda }{2}\left(\overset{L}{\underset{k=1}{\sum }}u_{n,k}^{2}+\overset{L}{\underset{k=1}{\sum }}v_{j,k}^{2}\right). \end{array}$$ (7)

To solve this loss function, stochastic gradient descent (SGD) is employed (Ahmadian et al., 2025; Lin X. et al., 2025; Lei et al., 2024). The method computes the gradient of the loss function with respect to the combined parameters and updates them in a descending direction:


$$\begin{array}{l} \{\begin{array}{c} u_{m,k}\leftarrow u_{m,k}-\eta \frac{\partial \varepsilon_{m,j}}{\partial u_{m,k}}\\ v_{j,k}\leftarrow v_{j,k}-\eta \frac{\partial \varepsilon_{m,j}}{\partial v_{j,k}} \end{array} \end{array}$$ (8)

From Equations 7 and 8, the partial derivative of the loss is derived:


$$\begin{array}{l} \begin{array}{l} u_{m,k}\leftarrow u_{m,k}+\eta v_{j,k}\left(f_{m,j}^{\prime }-\sum_{k=1}^{L}u_{m,k}v_{j,k}\right)-\lambda \eta u_{m,k},\\ v_{j,k}\leftarrow v_{j,k}+\eta u_{m,k}\left(f_{m,j}^{\prime }-\sum_{k=1}^{L}u_{m,k}v_{j,k}\right)-\lambda \eta v_{j,k}. \end{array} \end{array}$$ (9)

Here, η denotes the learning rate. *U* and *V* are optimized to minimize errors on the known values, yielding *R* = *UV*^*T*^. The error between the estimated and actual data is $err_{m,j}=f_{m,j}^{\prime }-\sum_{k=1}^{L}u_{m,k}v_{j,k}$, i.e.,


$$\begin{array}{l} \begin{array}{l} u_{m,k}\leftarrow u_{m,k}+\eta v_{j,k}err_{m,j}-\lambda \eta u_{m,k},\\ v_{j,k}\leftarrow v_{j,k}+\eta u_{m,k}err_{m,j}-\lambda \eta v_{j,k}. \end{array} \end{array}$$ (10)

#### 4.2.2 Online feature evaluation

The principal advantage of GA-OS^2^FS lies in its independence from missing value completion via an LFA model. The method sustains a feature subset whose fitness is evaluated through real classification accuracy. Consequently, the search process gains the capacity to tolerate and sidestep local misleading associations stemming from potential completion errors. As a prominent and widely implemented evolutionary optimization method, the GA offers several compelling advantages. GA maintains a diverse population of candidate solutions, enabling simultaneous exploration of multiple regions in the solution space. Through mechanisms such as selection, crossover, and mutation, it effectively combines and propagates beneficial gene patterns while continually introducing new variations. This population-based strategy significantly mitigates the risk of premature convergence to local optima, making GA particularly robust in navigating complex, multimodal search landscapes commonly encountered in feature selection. GA operates solely on the evaluation of candidate fitness, requiring no derivative information of the objective function. This characteristic renders it highly suitable for optimizing non-differentiable, discontinuous, or noisy objective functions—frequently the case in feature selection where the fitness is often a classification error rate or another performance metric derived from a learning model. The evolutionary process inherently promotes solutions that achieve an optimal balance between multiple, often competing, objectives. In feature selection, fitter individuals naturally tend to be those that maximize classification performance while minimizing the number of selected features, without needing an explicitly tuned regularization parameter. This emergent trade-off helps in discovering compact, discriminative feature subsets. The evaluation of fitness for each individual in a population is independent of others, making this computational step “embarrassingly parallel.” This allows for efficient distribution across multiple processors or cores, drastically reducing wall-clock time and enhancing the scalability of GA for large-scale or high-dimensional problems. Collectively, these advantages establish Genetic Algorithms as a powerful, flexible, and efficient metaheuristic framework for tackling the inherently combinatorial and complex problem of feature selection.

Given a dataset $D=\left(xi,y_{i}\right){i=1}^{m}$ with *m* samples and *n* features, where $x_{i}\in {\mathbb{R}}^{n}$ represents the feature vector and $y_{i}\in Y$ denotes the class label, the feature selection problem aims to identify an optimal subset of features S⊆1,2,…,n that maximizes classification performance while minimizing dimensionality. This binary optimization problem can be formulated as:


$$\begin{array}{l} \underset{b\in {\left\{0,1\right\}}^{n}}{\min}J\left(b\right)=\alpha \cdot \varepsilon \left(\text{X}⊙\left(1_{m}b^{T}\right),y\right)+\beta \cdot \frac{{∥b∥}_{0}}{n} \end{array}$$ (11)

where $b={\left[b_{1},b_{2},\ldots ,b_{n}\right]}^{⊤}$ is a binary vector with *b*_*j*_ = 1 if feature *j* is selected, and *b*_*j*_ = 0 otherwise, $|b|0=\sum {j=1}^{n}b_{j}$ denotes the ℓ_0_-norm counting selected features, *X* ∈ ℝ^*m* × *n*^ is the feature matrix with *Xij* = *xi, j*, E(·) represents the classification error function, α and β are weighting coefficients balancing classification accuracy and feature sparsity, ⊙ denotes element-wise multiplication, 1_*m*_ is an *m*-dimensional vector of ones.

Each candidate solution (chromosome) is encoded as a binary vector *b* ∈ 0, 1^*n*^. The initial population $P^{\left(0\right)}=b_{1},b_{2},\ldots ,b_{N}$ of size *N* is generated randomly:


$$\begin{array}{l} b_{ij}^{\left(0\right)}\sim Bernoulli\left(p_{0}\right),\forall i\in \left\{1,...,N\right\},\forall j\in \left\{1,...,n\right\} \end{array}$$ (12)

where *p*_0_ = 0.5 ensures unbiased initial exploration of the feature space.

The fitness evaluation metric is primarily assessed by measuring the classification error achieved using the selected features. As a wrapper-based method, this approach directly employs the performance of a target classifier—such as support vector machine (SVM)—to determine the quality of a candidate feature subset. The classification error serves as a direct and interpretable indicator of how well the selected features support the learning algorithm in discriminating between classes. Typically, to ensure robustness and prevent overfitting, the error is estimated via cross-validation or hold-out validation. This design aligns the feature selection process closely with the end classification task, thereby enhancing the relevance and discriminative power of the final feature subset.

To form the mating pool $M^{\left(t\right)}$ for generation *t*, individuals are selected probabilistically based on their fitness. The selection probability for chromosome *b*_*i*_ is:


$$\begin{array}{l} p_{i}^{sel}=\frac{w\left(f_{j}\right)}{\sum_{j=1}^{N}w\left(f_{j}\right)},w\left(f\right)=\frac{1}{f+\iota } \end{array}$$ (13)

Here, ι > 0 is a small constant preventing division by zero. The cumulative distribution function is:


$$\begin{array}{l} P_{i}^{sel}=\overset{i}{\underset{j=1}{\sum }}p_{j}^{sel},i=1,....,N \end{array}$$ (14)

For each selection, a random number *r* ~ *U*(0, 1) is generated, and chromosome *b*_*k*_ is selected where $k=\min i:P_{i}^{\text{sel}}\geq r$.

With probability *p*_*c*_, pairs of parent chromosomes undergo single-point crossover. For parents *b*_*p*_ and *b*_*q*_, a crossover point *c* is randomly selected:


$$\begin{array}{l} c\sim U\left\{1,2,...,n-1\right\} \end{array}$$ (15)

Two offspring $b_{p}^{\prime }$ and $b_{q}^{\prime }$ are generated as:


$$\begin{array}{l} \begin{array}{l} b_{p}^{\prime }=\left[b_{p,1},...,b_{p,c},b_{q,c+1},...,b_{q,n}\right]\\ b_{q}^{\prime }=\left[b_{q,1},...,b_{q,c},b_{p,c+1},...,b_{p,n}\right] \end{array} \end{array}$$ (16)

If crossover is not applied (with probability 1 − *p*_*c*_), the offspring are exact copies of the parents.

Each gene in the offspring undergoes mutation with probability *p*_*m*_. For gene *b*_*ij*_:


$$\begin{array}{l} b_{ij}^{\prime }=\{\begin{array}{ccc} 1-b_{ij} & \text{with probability} & p_{m}\;\\ b_{ij} & \text{with probability} & 1-p_{m} \end{array} \end{array}$$ (17)

This operator maintains population diversity and enables exploration of new regions in the search space.

To guarantee monotonic improvement across generations, the algorithm employs an elitism strategy. The best chromosome $b_{\text{best}}^{\left(t\right)}$ from generation *t* is preserved by replacing the worst chromosome in the offspring population $P^{\prime (t)}$:


$$\begin{array}{l} b_{worst}^{\prime }=b_{best}^{\left(t\right)},worst=\arg\underset{i}{\max}f_{i}^{\prime } \end{array}$$ (18)

This ensures that:


$$\begin{array}{l} f_{best}^{\left(t+1\right)}\leq f_{best}^{\left(t\right)},\forall t \end{array}$$ (19)

The genetic algorithm for OS^2^FS begins with inputs including the feature matrix *X* ∈ ℝ^*m* × *n*^, label vector *y* ∈ ℝ^*m*^, population size *N*, maximum iterations *T*_max_, crossover probability *p*_*c*_ (default: 0.8), mutation probability *p*_*m*_ (default: 0.05), mutation strength μ (default: 0.01), and hold-out folds *k* (default: 0). Initially, it sets *t* = 0 and generates the population $P^{\left(0\right)}$ via Equation 11, then evaluates each individual by computing $f_{i}=F\left(Xbi,y,k\right)$ for *i* = 1, …, *N*, and identifies the best individual as $b{\text{best}}^{\left(0\right)}=\arg\min b\in P^{\left(0\right)}f$ with fitness $f_{\text{best}}^{\left(0\right)}=\min f$. While *t* < *T*_max_, the algorithm performs selection to form the mating pool $M^{\left(t\right)}$ using roulette wheel selection (Equations 12, 13), generates offspring $P^{\prime (t)}$ via single-point crossover (Equations 14, 15) with probability *p*_*c*_, applies bit-flip mutation (Equation 16) to $P^{\prime (t)}$ with probability *p*_*m*_, and evaluates the offspring by computing $f_{i}^{\prime }=F\left(Xbi^{\prime },y,k\right)$. It updates the best individual if $\min f_{i}^{\prime }<f{\text{best}}^{\left(t\right)}$, then applies elitism by replacing the worst individual in $P^{\prime (t)}$ with *b*best^(*t*)^ (Equation 17), followed by updating $P^{\left(t+1\right)}=P^{\prime (t)}$, *f*^(*t* + 1)^ = *f*′, and the convergence curve $c_{t+1}=f_{\text{best}}^{\left(t\right)}$, and incrementing *t* = *t* + 1. After the loop, it extracts the results: $Sf=j:b{\text{best},j}^{\left(T_{\max}\right)}=1,j=1,\ldots ,n$, $n_{f}=|Sf|$, $X\text{selected}=X_{:,Sf}$, and returns $\left(X\text{selected},S_{f},n_{f},c\right)$ as output. Redundancy analysis is first performed on individual features using Markov, and then on the selected features using Equation 1.

The algorithm's convergence is guaranteed by the elitism strategy, which ensures the best fitness value is non-increasing:

**Theorem 1 (Monotonic Convergence)**. For the GA-OS^2^FS algorithm with elitism, the sequence of best fitness values $f_{\text{best}}^{\left(t\right)}{t=0}^{T\max}$ is monotonically non-increasing.

*Proof*. By construction, the elitism strategy preserves the best solution from generation *t* in generation *t* + 1. Therefore, $f_{\text{best}}^{\left(t+1\right)}\leq f_{\text{best}}^{\left(t\right)}$ for all *t*.

The expected time complexity per iteration is $O\left(N\cdot \left(n+C_{F}\right)\right)$, where $C_{F}$ is the cost of evaluating the fitness function for one chromosome. The overall complexity for *T*_max_ iterations is $O\left(T_{\max}\cdot N\cdot \left(n+C_{F}\right)\right)$.

The proposed GA-OS^2^FS algorithm provides an effective approach for streaming feature selection, combining the global search capability of genetic algorithms with direct performance evaluation using the target classifier.

## 5 Experiments

### 5.1 General settings

#### 5.1.1 Datasets

This section presents the experimental evaluation conducted on six real-world datasets obtained from two key sources: DNA microarray repositories and the benchmark collection from the Neural Information Processing Systems (NIPS) 2003 conference. These datasets are widely recognized in the machine learning and bioinformatics communities for assessing feature selection and classification algorithms under high-dimensional, small-sample conditions. The inclusion of microarray data ensures the examination of genetic expression patterns, while the NIPS 2003 datasets provide a diverse range of problem domains and complexity levels, thereby enabling a comprehensive analysis of the proposed method's robustness and generalizability. A detailed summary of the datasets—including the number of features, samples, and classes—is provided in Table 1 for reference.

**Table 1:** Details of the datasets.

**Mark**	**Dataset**	**Features**	**Instances**	**Class**
D1	USPS	242	1,500	2
D2	Madelon	501	2,600	6
D3	COIL20	1,025	1,440	20
D4	Colon	2,001	62	2
D5	Lung	3,313	83	5
D6	DriveFace	6,401	606	3

#### 5.1.2 Baselines

To comprehensively evaluate the efficacy of the proposed model, a rigorous comparative analysis is conducted against four state-of-the-art online streaming feature selection (OS^2^FS) methods, which are recognized as established benchmarks in the field. The selected competitors include Fast-OSFS, SAOLA, and LOS-SA. This diverse set of algorithms encompasses various strategic approaches to handling feature streams—such as leveraging pairwise feature relations, redundancy analysis, and sparsity-aware selection—thereby ensuring a robust and multifaceted comparison. Furthermore, to objectively assess the quality of the feature subsets selected by each method, the evaluation employs three fundamental yet powerful classifiers: Support Vector Machine (SVM), k-Nearest Neighbors (KNN), and Random Forest (RF). These classifiers were chosen for their distinct learning mechanisms: SVM seeks optimal separating hyperplanes, KNN relies on local similarity, and RF utilizes ensemble decision-making. Their combined use helps verify whether the selected features generalize well across different inductive biases and are not tailored to a single classification model.

Detailed parameter configurations for all compared OS^2^FS algorithms and the three classifiers are systematically summarized in Tables 2, 3, respectively, to ensure full reproducibility of the experiments. All algorithms are implemented in MATLAB to maintain a consistent computational environment. And all experiments utilize five-fold cross-validation, meaning each dataset is randomly divided into an 80% training portion and a complementary 20% test portion. To account for randomness in data partitioning and algorithm initialization, each dataset is executed 10 times; the final reported result is the average predictive accuracy across these runs, along with its standard deviation where applicable.

**Table 2:** Algorithm parameters.

**Mark**	**Algorithm**	**Parameter**
M1	GA-OS^2^FS	Z test, Alpha is 0.05.
M2	LOSSA	Z test, Alpha is 0.05. (TSMC, 2022)
M3	Fast-OSFS	Z test, Alpha is 0.05. (TPAMI, 2013)
M4	SAOLA	Z test, Alpha is 0.05. (TKDD, 2016)
M5	SFS-FI	Z test, Alpha is 0.05. (TNNLS, 2021)

**Table 3:** Details of the classifiers.

**Classifier**	**Parameter**
KNN	The number of neighbors was set to 3.
Random forest	6 decision trees.
CART	Predefined parameter settings.

All trials were conducted on a standard personal computer equipped with an Intel Core i7 processor running at 2.40 GHz and 16 GB of RAM, ensuring that the computational demands of the online feature selection and classification processes were feasibly met within a common research setup. This controlled hardware environment also aids in the fair comparison of runtime and efficiency where relevant.

#### 5.1.3 Experimental configuration

The efficacy of the GA-OS^2^FS model is rigorously assessed by benchmarking it against the aforementioned suite of advanced algorithms, specifically within the challenging context of sparse streaming features. This scenario is deliberately chosen to simulate real-world conditions where data incompleteness and sequential feature arrival are prevalent, thereby testing the models' robustness and adaptability. To ensure a fair and statistically grounded comparison of performance across all algorithms, a non-parametric Friedman test is conducted at a stringent 95% confidence level. This test is employed under the null hypothesis that all algorithms perform equivalently, providing a holistic view of performance rankings across multiple datasets.

Furthermore, to drill down into pairwise performance differences, a paired Wilcoxon signed-rank test is applied at a 0.1 significance level. This test is specifically designed to examine whether the observed performance differences between the GA-OS^2^FS model and each individual baseline algorithm are statistically significant, rather than attributable to random chance. The resulting p-values from this comprehensive statistical analysis are consistently below the significance threshold. This robust statistical evidence leads to the conclusive finding that the GA-OS^2^FS model significantly and consistently outperforms all competing algorithms in the evaluation, demonstrating its superior capability in selecting informative features from sparse, evolving data streams.

### 5.2 Accuracy comparison

#### 5.2.1 Detailed analysis under 10% missing data rate

**Figure 1:**
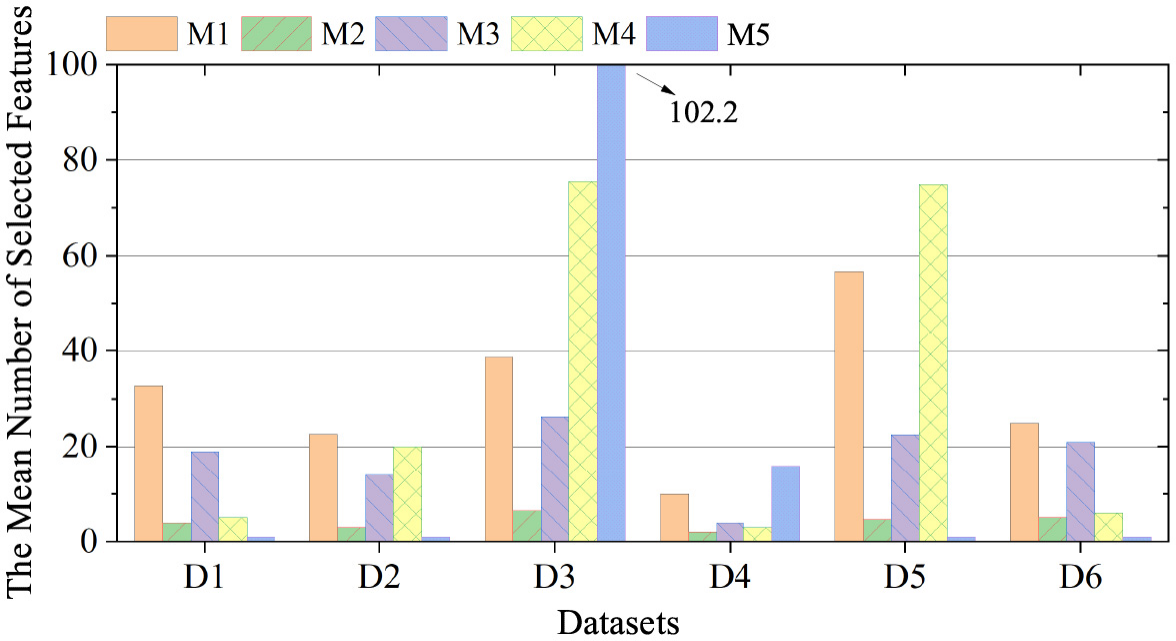
The mean number of selected features.

To investigate the impact of missing data on feature selection performance, a missing-at-random scenario with a 10% data loss rate is established as a representative and practically relevant case for detailed analysis. This specific rate is chosen to simulate a common yet challenging level of data incompleteness encountered in real-world streaming applications. As illustrated in Figure 1, the average number of features selected by each compared method under this sparse condition is presented. The bar chart reveals distinct strategies among the algorithms: some methods maintain a conservative, highly selective profile, while others retain a larger fraction of the feature stream, reflecting different trade-offs between redundancy elimination and information preservation.

Correspondingly, Table 4 documents the concrete predictive performance outcomes, quantified by classification accuracy, when applying three fundamentally different classifiers—K-Nearest Neighbors (KNN), Support Vector Machine (SVM), and Random Forest (RF)—to the feature subsets identified by each method. This multi-classifier evaluation is crucial, as it demonstrates whether the selected features provide robust discriminative power independent of a specific learning algorithm's bias. The results in the table allow for a direct, quantitative comparison of how the parsimony or comprehensiveness of a selected feature subset, as shown in Figure 1, ultimately translates into generalization accuracy across diverse classifiers. This integrated analysis of subset size (Figure 1) and classification efficacy (Table 4) provides a comprehensive view of each algorithm's effectiveness in balancing feature reduction with predictive performance under the specified missing data condition.

**Table 4:** The accuracy when the missing rate is 0.1.

**M/D**	**D1**	**D2**	**D3**	**D4**	**D5**	**D6**
M1	90.79 ± 0.87	59.14 ± 1.36	92.62 ± 1.13	81.77 ± 3.48	89.47 ± 1.31	94.91 ± 0.50
M2	84.33 ± 0.64	54.97 ± 0.69	84.87 ± 2.63	80.45 ± 2.59	84.79 ± 2.77	92.29 ± 0.58
M3	85.48 ± 0.63	54.83 ± 0.97	71.08 ± 2.22	78.88 ± 2.59	84.40 ± 2.43	93.14 ± 0.67
M4	80.18 ± 0.54	53.79 ± 0.80	88.59 ± 0.49	77.65 ± 3.19	83.38 ± 2.32	93.34 ± 0.71
M5	72.18 ± 0.54	49.33 ± 0.54	80.56 ± 3.48	78.63 ± 2.66	62.49 ± 2.96	85.51 ± 1.02

##### 5.2.1.1 Statistical significance (Friedman test)

To statistically validate the performance differences observed among the compared algorithms under the 10% missing data scenario, a non-parametric Friedman test was conducted across all datasets. The test returned a *P*-value of 0.0011, which is substantially below the commonly adopted significance threshold of 0.05. This very small *P*-value allows us to firmly reject the null hypothesis that all algorithms perform equally. Therefore, the results provide strong statistical evidence that there are significant differences in the overall performance ranks of the evaluated methods. More specifically, the outcome underscores that the proposed GA-OS^2^FS model achieves a distinctly superior ranking compared to the alternative algorithms, confirming its enhanced robustness and effectiveness when handling incomplete streaming features with 10% missing values. Such a statistically significant finding further reinforces the practical relevance and reliability of the GA-OS^2^FS approach in real-world sparse data environments.

##### 5.2.1.2 Analysis of feature selection quantity

The GA-OS^2^FS model demonstrates stable feature selection across different sparse datasets. In contrast, algorithms like SAOLA show considerable variation in the number of features selected depending on the dataset. Key observations include:

1. An intriguing pattern observed in the experiments is that several compared algorithms tend to select a considerably larger set of features, yet consistently deliver lower classification accuracy compared to the GA-OS^2^FS model. This indicates that simply retaining more features does not guarantee better predictive performance, and often points to insufficient or less effective redundancy analysis in the feature selection process. When redundancy is not adequately assessed, many retained features may be non-informative, noisy, or highly correlated with one another, thereby adding little discriminative value while increasing model complexity and the risk of overfitting. In contrast, the GA-OS^2^FS model appears to implement a more refined mechanism for evaluating feature relevance and redundancy, enabling it to identify and retain a compact yet highly informative subset of features that better supports accurate classification.
2. Other algorithms, such as SFS-FI, occasionally select an extremely small number of features—in some cases as few as only one—on particular datasets. This behavior is likely attributable to their limited ability to comprehensively capture all essential features when processing incomplete data streams. Specifically, these methods may prematurely converge on the first few features that appear sufficiently relevant, while failing to adequately evaluate or retain subsequently arriving features that are equally or more informative. As a result, they miss critical feature interactions and discard valuable discriminative information, ultimately leading to suboptimal classification performance due to an oversimplified and incomplete feature subset.
3. The GA-OS^2^FOS model performs comprehensive relevance and redundancy analysis through a structured genetic optimization process. By leveraging GA-based feature evaluation, it dynamically assesses each feature's discriminative power and mutual dependencies within the evolving stream. This enables the model to systematically identify and retain truly informative features while filtering out redundant or noisy ones. Consequently, it avoids the premature discarding of important predictive information—a common pitfall in many streaming feature selection methods. As a result, the model consistently achieves high classification accuracy while maintaining a compact and efficient feature subset, effectively balancing model simplicity with representational completeness.

##### 5.2.1.3 Classification performance

As shown in Table 4, the GA-OS^2^FS model exceeds the performance of its rivals on six datasets. Key observations include:

1. GA-OS^2^FS vs. Fast-OSFS: the experimental results demonstrate that the GA-OS^2^FOS model consistently delivers superior classification accuracy across a majority of the benchmark datasets. In contrast, the Fast-OSFS algorithm exhibits notable limitations. Its performance is constrained by a reliance on zero-imputation to handle incomplete data—a method that simply fills missing values with zeros. While straightforward, this approach fails to capture any underlying data structure or relationships, potentially distorting the feature space. Furthermore, Fast-OSFS employs a less comprehensive analysis of feature relevance and redundancy. This dual shortcoming—crude data imputation coupled with insufficient feature evaluation—often results in the misclassification of features during the streaming selection process. Informative features may be incorrectly discarded, while redundant or noisy ones might be retained. Consequently, these limitations fundamentally undermine the quality of the final selected feature subset, leading to its comparatively poorer predictive performance.
2. GA-OS^2^FS vs. SAOLA: the SAOLA algorithm operates primarily by assessing pairwise relationships between features, evaluating them in isolation or through limited local comparisons. While efficient, this approach may overlook more complex, higher-order interactions among feature subsets, and its incremental update mechanism can be sensitive to the arrival order of features in a stream. In contrast, the proposed GA-OS^2^FS model integrates and fully leverages the complementary strengths of the LFA model and the GA framework. LFA assists in capturing underlying low-rank structures and global correlations even under sparse or missing data conditions, while GA performs robust, population-based search to dynamically evaluate and retain the most discriminative feature combinations. This hybrid strategy enables GA-OS^2^FS to consistently identify critical features in real-time from evolving data streams, without being constrained by purely local or pairwise evaluations.
3. GA-OS^2^FS vs. SFS-FI: sparse streaming data often loses critical feature interactions, which severely challenges methods like SFS-FI that rely on detecting these dependencies. Unable to accurately assess feature relevance under sparsity, SFS-FI tends to select redundant or omit informative features, resulting in the lowest classification accuracy in evaluations. This underscores its limited robustness with incomplete data and highlights the advantage of GA-OS^2^FS's more resilient design.
4. Among the evaluated models, LOSSA achieves the second-highest classification accuracy after GA-OS^2^FS when processing completed sparse streaming features, demonstrating the benefit of using Latent Factor Analysis (LFA) for data completion. However, LOSSA relies on conventional relevance and redundancy analyses, which lack adaptability to capture complex feature interactions or evolving stream characteristics, limiting its average accuracy. In contrast, GA-OS^2^FS integrates a Genetic Algorithm strategy, performing a global, population-based search that evaluates multiple feature subsets and iteratively refines them using crossover, mutation, and fitness feedback. This enables GA-OS^2^FS to discover more discriminative feature combinations, leading to superior predictive performance and offering a more adaptive solution for accurate feature selection in sparse streaming environments.

##### 5.2.1.4 The Wilcoxon signed-ranks test

To rigorously substantiate the statistically significant superiority of the proposed GA-OS^2^FS algorithm, a non-parametric Wilcoxon signed-rank test was employed. This test was specifically chosen for its appropriateness in comparing the performance of two related samples—in this case, the paired average classification accuracy values of the GA-OS^2^FS model against each of the benchmark methods across multiple datasets. The detailed outcomes of these pairwise comparisons, including the calculated test statistics and corresponding *P*-values, are comprehensively presented in Table 5.

**Table 5:** The rank sum of the Wilcoxon signed-ranks.

**M1 vs. Others**	**R+^a^**	**R-^a^**	***P*-values^b^**
M2	21	0	0.0156
M3	21	0	0.0156
M4	21	0	0.0156
M5	21	0	0.0156

^a^A larger value denotes a higher accuracy.

^b^There is no significant difference when *P*-values ∈ [0.1, 0.9] at the 0.1 significance level.

The statistical analysis yields a clear and robust conclusion: even at a missing data rate of 0.1—representing a modest yet realistic level of data incompleteness–the GA-OS^2^FS approach demonstrates a consistent and statistically significant performance advantage. It reliably outperforms the alternative algorithms on a substantial majority of the evaluated datasets. This early and significant lead established by GA-OS^2^FS under sparse conditions highlights its inherent robustness and effective design for handling incomplete data streams from the outset.

In summary, relative to traditional OS^2^FS models, completing sparse streaming features via the LFA model generally minimizes information loss and enhances overall results. Consequently, both GA-OS^2^FS and LOSSA deliver the strongest performance on sparse streaming data. Nevertheless, the feature subsets selected by GA-OS^2^FS yield higher classification accuracy than those from LOSSA, demonstrating that GA can improve the accuracy of feature selection.

#### 5.2.2 Accuracy analysis with higher missing rates

**Figure 2:**
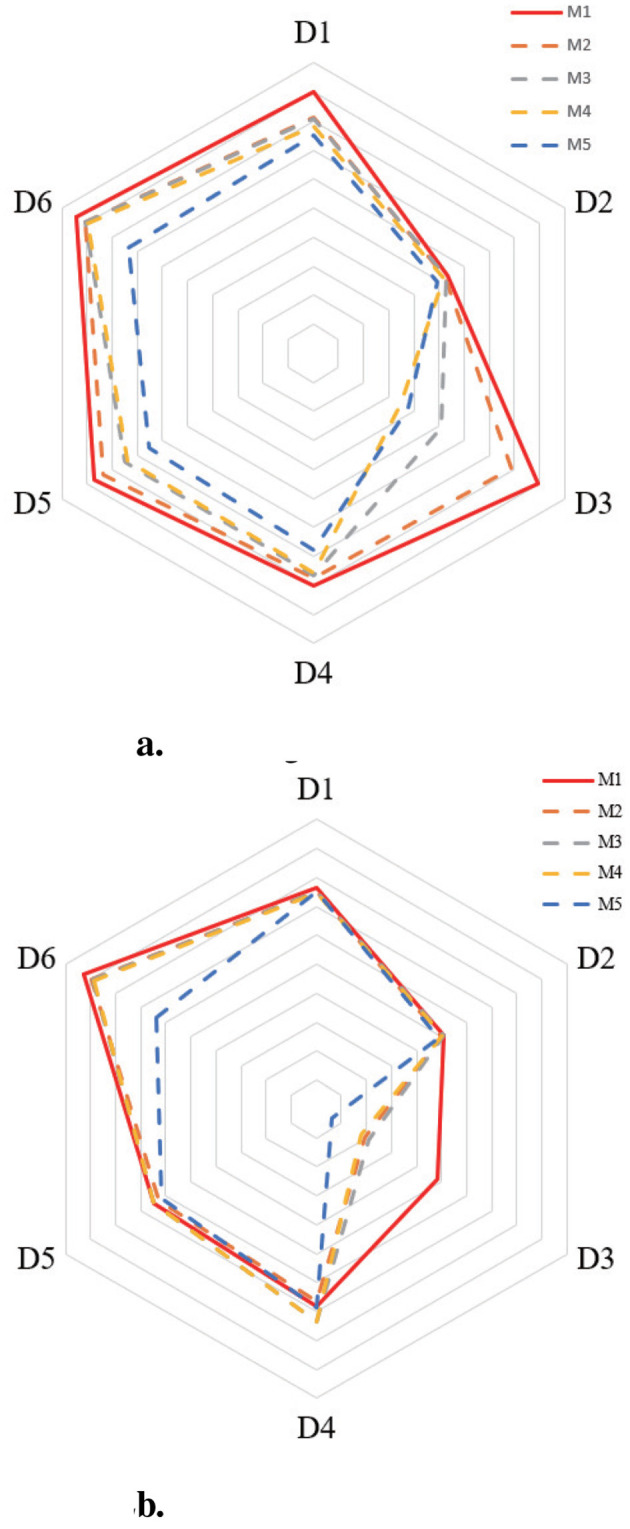
The accuracy analysis with higher missing rates. **(a)** Missing data rate is 0.5. **(b)** Missing data rate is 0.9.

This study evaluates the effectiveness of the 3WDO model by comparing it against six prominent OS^2^FS models—Fast-OSFS, SAOLA, SFS-FI, and LOSSA—across six datasets under missing data rates ranging from 0.5 to 0.9. While LOSSA is designed to handle missing values, the other three baseline algorithms are oriented toward complete feature streams. To adapt them for sparse data, zero-imputation is applied to fill missing entries for Fast-OSFS, SAOLA, and SFS-FI. Results are highlighted where any algorithm demonstrates superior performance compared to the others. Table 6 provides a pairwise comparison between GA-OS^2^FS and each baseline using the Wilcoxon signed-ranks test. The average accuracy trends of all models on datasets D1-D4 are visualized in Figure 2.

**Table 6:** The rank sum of the the Wilcoxon signed-rank test on OSFS and OS^2^FS models.

**ρ**	**M2**		**M3**		**M4**		**M5**	
	**R+** ^a^	**R-** ^a^	**R+** ^a^	**R-** ^a^	**R+** ^a^	**R-** ^a^	**R+** ^a^	**R-** ^a^
0.5	21	0	21	0	21	0	21	0
0.9	21	0	16	0	16	0	20	1

^*a*^denotes missing data rate.

##### 5.2.2.1 Overall accuracy of the GA-OS^2^FS model

Across all benchmark datasets examined, the average classification accuracy of the GA-OS^2^FS model demonstrates a gradual yet consistent decline as the missing data rate increases. This overall trend aligns with expectations, as higher rates of missing entries inevitably compromise the informational integrity of the feature stream, making it more challenging to reliably identify and retain discriminative features. Notably, however, on several specific datasets, the model's accuracy exhibits only minor fluctuations—remaining relatively stable even as the missing rate rises. This suggests that the GA-OS^2^FS approach maintains a notable degree of robustness in certain data environments, likely due to its effective integration of latent factor completion and evolutionary search, which together help preserve critical predictive information under moderate to high sparsity conditions.

##### 5.2.2.2 Wilcoxon signed-rank test results

Table 6 presents the Wilcoxon signed-rank test results comparing the average accuracy of GA-OS^2^FS against other methods. The findings indicate that as the missing data rate increases from 0.5 to 0.9, the proposed algorithm outperforms most baseline methods on the majority of datasets.

##### 5.2.2.3 Performance on datasets

Observations from Figure 2 lead to the following conclusions:

1. For the majority of the evaluated algorithms, classification accuracy exhibits a progressive decline as the rate of missing data increases. This decline can be attributed to the growing incompleteness of the feature stream, which hinders the reliable assessment of feature relevance and redundancy. In contrast, the proposed GA-OS^2^FS model consistently achieves superior accuracy across most benchmark datasets, maintaining a clear performance advantage even as the missing data rate escalates from 0.5 to 0.9. This robustness stems from its integrated use of latent factor analysis (LFA) for structured data completion and genetic algorithm (GA)-guided feature optimization, which together preserve discriminative information and adaptively select informative features under sparse conditions. By comparison, conventional methods such as Fast-OSFS, SAOLA, and SFS-FI rely primarily on zero-filling (zero-imputation) to handle incomplete streaming features. While computationally simple, this approach substitutes missing entries with zeros—a strategy that distorts the original data distribution, disrupts inherent feature correlations, and often introduces artificial noise. Consequently, these methods are prone to selecting uninformative or redundant features, which undermines their classification performance and explains their significantly poorer results relative to the GA-OS^2^FS framework, especially under higher missing-rate scenarios.
2. For missing rates between 0.1 and 0.5, LOSSA generally achieves higher accuracy than baselines like Fast-OSFS, SAOLA, and SFS-FI, due to its LFA-based data completion providing better estimates than simple imputation (e.g., zero-filling). However, as missing data increases, limited known entries raise LFA's estimation error. This distorts the recovered feature space, causing relevant features to be misclassified as irrelevant and discarded, degrading selection quality. To address this, GA-OS^2^FS employs a genetic algorithm to partition and evaluate features more robustly. This enables a global, resilient importance assessment that is less sensitive to local completion errors. By reducing feature misclassification, it retains a more discriminative subset, yielding consistently higher accuracy than LOSSA and other baselines, especially as sparsity grows.

In summary, by pre-estimating missing data via the LFA and GA model, the GA-OS^2^FS model enhances the accuracy of traditional OS^2^FS approaches.

## 6 Conclusions

This study introduces GA-OS^2^FS, a novel uncertainty-aware framework for Online Sparse Streaming Feature Selection, designed to address critical shortcomings in conventional approaches. The framework innovatively integrates Genetic Algorithms (GA) to navigate the complex search space of dynamic feature subsets. GA-OS^2^FS operates through a synergistic two-component architecture: firstly, a Latent Factor Analysis (LFA) model that performs robust, dynamic imputation and reconstruction of inherently sparse and incomplete data matrices in real-time; secondly, a GA-based optimization mechanism that drives an intelligent, evolutionary search for discriminative features, effectively evaluating feature importance and interactions under uncertainty. Extensive empirical evaluation conducted across 10 diverse real-world datasets—spanning various domains and data characteristics—demonstrates that GA-OS^2^FS consistently surpasses state-of-the-art OSFS and OS^2^FS benchmarks. It achieves superior performance not only in selection accuracy and robustness but also in maintaining operational stability, all while ensuring computational efficiency. These results collectively underscore the framework's strong potential and adaptability for reliable, real-time feature selection in challenging high-dimensional streaming data environments.

Looking ahead, future research will concentrate on advancing the theory and practice of feature quality assessment within non-stationary streaming contexts. A primary direction involves refining and extending the evolutionary computation core, leveraging advanced Genetic Algorithm strategies and other meta-heuristics to develop more adaptive feature evaluation criteria and dynamic fitness functions. These innovations will be specifically tailored to track and respond to shifting data distributions. Furthermore, we will investigate efficient, dedicated techniques to manage concept drift, such as sophisticated incremental model update protocols and adaptive sliding window mechanisms. Additional promising avenues include exploring ensemble-based feature selection tactics that combine multiple selectors, and developing dynamic feature weighting schemes to continuously prioritize the most relevant features. The overarching goal of these endeavors is to significantly enhance the framework's responsiveness, resilience, and scalability when confronted with the evolving patterns of complex, real-world streaming applications.

## Data Availability

The original contributions presented in the study are included in the article/supplementary material, further inquiries can be directed to the corresponding author.
